# A preliminary trial examining a ‘real world’ approach for increasing physical activity among breast cancer survivors: findings from project MOVE

**DOI:** 10.1186/s12885-019-5470-2

**Published:** 2019-03-27

**Authors:** Cristina M. Caperchione, Catherine M. Sabiston, Sean Stolp, Joan L. Bottorff, Kristin L. Campbell, Neil D. Eves, Susan L. Ellard, Carolyn Gotay, Paul Sharp, Tanya Pullen, Kayla M. Fitzpatrick

**Affiliations:** 1 Faculty of Health, Human Performance Research Centre, University of Technology Sydney, Sydney, New South Wales Australia; 2 Institute for Healthy Living and Chronic Disease Prevention, University of British Columbia, Kelowna, British Columbia Canada; 3 School of Health and Exercise Science, University of British Columbia, Kelowna, British Columbia Canada; 4 Faculty of Physical Education, University of Toronto, Toronto, Ontario Canada; 5 School of Nursing, University of British Columbia, Kelowna, British Columbia Canada; 6 Faculty of Health Sciences, Australian Catholic University, Melbourne, Victoria Australia; 7 Department of Physical Therapy, University of British Columbia, Vancouver, British Columbia Canada; 8 Centre for Heart, Lung and Vascular Health, University of British Columbia, Kelowna, British Columbia Canada; 9 Cancer Centre of the Southern Interior, British Columbia Cancer Agency, Kelowna, British Columbia Canada; 10 School of Population and Public Health, University of British Columbia, Vancouver, British Columbia Canada

**Keywords:** Women, Breast cancer survivors, Oncology care, Physical activity, Microgrants, Financial incentives, Community-based intervention

## Abstract

**Background:**

Physical activity (PA) is a safe and effective strategy to help mitigate health challenges associated with breast cancer (BC) survivorship. However, the majority of BC survivors are not meeting the minimum recommended PA (≥150 min of moderate to vigorous intensity). Project MOVE was developed as a model for increasing PA that combined a) Microgrants: funds ($2000) awarded to applicant groups to develop and implement a PA initiative and b) Financial incentives: a reward ($500) for increasing group PA. The purpose of this paper was to provide an exploratory analysis of effectiveness of Project MOVE on PA behavior, PA motivation, and quality of life (QoL) in female BC survivors. The differential outcomes between women meeting and not meeting PA guidelines were also investigated.

**Methods:**

This pre-post test, preliminary trial included groups of adult (18+ years) self-identified female BC survivors, who were post-surgery and primary systemic chemo- and radiation therapy, and living in British Columbia, Canada. PA was assessed by accelerometry. PA motivation and QoL were assessed by self-report. Data were collected at baseline, 6-months, and 12-month time points. Repeated measures mixed ANOVAs were used to test changes in the main outcomes.

**Results:**

A total of 10 groups were awarded microgrants between May 2015 and January 2016. Groups comprised of 8 to 12 women with a total of 87 participants. A statistically significant increase was found between time points on weekly moderate to vigorous PA (*p* = .012). This was mediated by a significant interaction between those meeting PA guidelines and those not meeting guidelines at baseline by time points (*p* = .004), with those not meeting guidelines at baseline showing the greatest increase in MVPA. A statistically significant difference across time points was found for intrinsic motivation (*p* = .02), physical functioning (*p* < .001), physical health limitations (*p* = .001), emotional health limitations (*p* = .023), social functioning (*p* = .001) and general health (*p* = .004).

**Conclusion:**

These results provide promising support for a unique approach to increasing PA among BC survivors by empowering women and optimizing PA experiences through the use of microgrants and financial incentives.

**Trial registration:**

ClinicalTrials.gov NCT03548636, Retrospectively registered June 7, 2018.

## Background

Breast cancer (BC) is the most frequently diagnosed cancer and the second leading cause of cancer death among women worldwide [1]. In Canada, it is estimated that one in every nine women will be diagnosed with BC and approximately 5000 women will die from BC each year, representing 13% of all cancer deaths among women [2]. With advancements in early detection technology and improved treatment strategies, BC related mortality rates are declining, resulting in five year survival rates reaching 87% [2]. However, cancer treatments (i.e., surgery, chemotherapy, hormone therapy, and/or radiation) can result in long-term detrimental side effects including morbidity, decline in functional status, disability, and/or subsequent mental health sequelae [3, 4]. Physical activity (PA) is a safe, effective, and feasible intervention strategy that can help mitigate these effects [5–8] and is associated with numerous other health benefits among BC survivors, including weight loss or maintenance, reduced depression and anxiety, management of post-treatment symptoms, and improved social support and quality of life (QoL) [8–10]. Moreover, meeting or exceeding Canadian recommended aerobic PA guidelines of ≥150 min of moderate to vigorous PA (MVPA) per week has been associated with a reduction in BC recurrence and all-cause mortality [11]. However, PA levels are generally low among BC survivors, with up to 70% of BC survivors not meeting the minimum recommended guidelines [12–14]. Innovative strategies focused on ways to increase PA levels and subsequently improve post-diagnosis health and QoL of BC survivors, are required.

Project MOVE is an approach which combines the use of microgrants (small amount of funds awarded to community-based applicants to develop/implement a PA initiative) and financial incentives to prompt and sustain PA, while promoting a sense of empowerment and ownership of the program, provides a unique model aimed at increasing PA in this population. Although unique to cancer care research the few studies that have utilized microgrants as a catalyst for promoting health initiatives have reported success in raising awareness about the benefits of PA, as well as enabling and supporting women’s engagement in PA [15–18]. However, previous research has not examined behavior change nor assess the inclusion of additional tools, such as financial incentives, as a complementary strategy for increasing PA motivation. The overarching aim of the Project MOVE study was to evaluate the feasibility of this unique model, and explore the effectiveness of the model in terms of PA behavior, PA motivation, and QoL. Outcomes regarding the feasibility of Project MOVE have been published elsewhere [19, 20]. The purpose of this paper was to provide an exploratory analysis of effectiveness on PA behavior, PA motivation and QoL. Additionally, we aimed to investigate differential effects between those meeting and not meeting PA guidelines on these outcome variables.

## Methods

### Study design

Project MOVE’s trial rationale and protocol have been described elsewhere [21]. In brief, this study is based on a quasi-experimental pre-post design. Baseline, six-month and one-year follow-up measures were undertaken between May 2015 and March 2017. Minor deviations were made from the original protocol due to the pragmatic nature of this trial. For instance, the analytical framework was modified and some of the findings (e.g., social support) have been reported elsewhere [19, 20]. Informed written consent was obtained prior to baseline assessments from all participants. This study was approved by the Behavioural Research Ethics Board at the University of British Columbia (#H14–02502).

### Participants, recruitment and eligibility

For the purpose of this study, a survivor is defined based on the National Coalition for Cancer Survivorship as someone who has lived with, through and beyond a cancer diagnosis [22]. Groups (8–12 per group) of women (18+ years) who self-defined as BC survivors living in the Okanagan region of British Columbia, Canada were eligible to participate. Based on interests from women to engage in physical activity with both other survivors and healthy women of similar ages, Project MOVE team members adjusted the recruitment eligibility during the initial recruitment phase so that groups comprised of at least 50% BC survivors were eligible. Women living in the Okanagan who wished to participate but were not BC survivors were eligible providing there was space in the groups after all interested BC survivors were accommodated.

A variety of recruitment techniques were employed, including meetings with cancer -related community organizations (e.g., BC Cancer Agency), a project specific website, news items in the local media (e.g., newspapers, online forums) social media announcements (Facebook and Twitter), posters distributed to local businesses, community centers and medical clinics and attendance at the annual Run for the Cure fundraising event.

### Project MOVE model

There was no pre-determined initiative (aka “intervention”) promoted or developed by the researchers. Instead each applicant group was invited to design its own PA intervention and apply for up to $2000 to enable access to facilities, equipment, tools and resources, instruction and/or transportation to implement the intervention. Groups were encouraged to contact members of the research team if they needed support throughout the application process to help conceptualize their project.

For the application process, each group designated a leader who acted as the primary contact and was responsible for submitting an online microgrant application. Each group leader submitted an application with details regarding the PA their group planned to do each week, how this activity would contribute to increasing the group’s overall PA levels, and outline a proposed budget and timeline. All submitted applications were initially screened for eligibility. Those deemed eligible were then processed and further evaluated by a Grant Review Panel consisting of three members from the research team, a representative from the Canadian Cancer Society and a local BC survivor who was not part of a group submitting a microgrant application. Successful applicant groups were notified by email and were informed of program obligations (i.e., participate in data collection, provide final report). Unsuccessful applicants were also notified and provided feedback outlining reasons for the not funded decision. Each group that received a microgrant was also informed that if they increased their group’s mean PA at the 6 month follow-up (assessed via accelerometry), they would receive an additional $500 incentive.

### Outcome measures

Demographic data were collected at baseline and all other data (i.e., BC information, PA behavior, PA motivation, and QoL) were collected at baseline, 6 months and 12 months, at a location and time convenient for each Project MOVE group (e.g., community center, local fitness center). At these data collection sessions, participants were given accelerometers and accelerometer log sheets, instructed on how to use each item, and asked that the accelerometers and log sheets be returned to their group’s primary contact person after the required wear time. Members of the research team were available to provide instructions and answer participant’s questions during data collection. Participants who were unable to attend the group measurement session were followed up by a research team member to schedule an alternative location (e.g., participants home) and time to complete measurements and receive an accelerometer. All measures were undertaken by two research team members, who collected all data at each time point.

#### Demographics and BC information

Demographic variables included age, education, ethnicity, employment and marital status. Additional information related to BC included date of most recent diagnosis, stage of BC at diagnosis, type of treatment, date of last treatment received and menopausal status.

#### Physical activity

PA was assessed objectively using an Actigraph GT3X™ accelerometer (ActiGraph, Pensacola, FL) worn on the hip during all waking hours over seven days. The accelerometers were initialized to collect acceleration counts in tri-axial mode and data were aggregated to 60-s epoch [23, 24] using Actilife software version 6.13.2. Daily measures of MVPA (> 1951 cpm) and wear time were extracted, based on activity counts per minute [24]. Non-wear time was defined as 60 min of consecutive zero counts, and included a 2 min spike tolerance of 50 cpm of movement. Valid wear time was defined as ≥10 h on ≥5 days, within the 7-day period at baseline, 6-months, and 12-months. Accelerometer data were used to indicate minutes of MVPA [25, 26]. Baseline accelerometer data were also used to classify participants as meeting or not meeting 150 min of weekly MVPA [26–28].

#### Physical activity motivation

Motivation to engage in PA was assessed using the Behavioral Regulation in Exercise Questionnaire- version 3 (BREQ-3). The valid and reliable, 24 item instrument [29, 30] measures amotivation (e.g., “I can’t be bothered to exercise”), external regulation (e.g., “I exercise because other people say I should”), introjected regulation (e.g., “I feel guilty when I don’t exercise”), identified regulation (e.g., “I value the benefits of exercise”), integrated regulation (e.g., “I consider exercise to be part of my identity”), and intrinsic regulation (e.g., “I exercise because it’s fun”) of exercise behavior based on Deci & Ryan’s [31, 32] continuum of self-determined motivation. Participant responses were scored as the average of each of the items theoretically proposed to be measuring each aspect of behavioral regulation [33].

#### Quality of life

Quality of life was assessed using relevant subscales from the valid and reliable SF 36 Medical Outcomes Study Survey (SF-36/RAND 36) [34, 35]. These subscales included physical functioning, role limitations due to physical health problems, role limitations due to personal or emotional problems, social functioning, and general health perceptions. All items were then scored on a 0 to 100 range using pre-coded numeric values, with a high score representing a more favorable health state. Additionally, items in each of the five domains were averaged together to create eight separate domain scores [36].

### Statistical analysis

All statistical analyses were conducted using SPSS version 22. Descriptive analyses were completed and presented as means and standard deviations (SD) for continuous variables and as frequencies and proportions for categorical data. Intention-to-treat analyses were conducted with the last known measurement carried forward for those who dropped out. Baseline accelerometer measures for participants who were outliers, or accumulated insufficient wear time at 6-month and 12-month follow-up were carried forward. Those with outlier measurements, as defined by +/− three standard deviations from the mean, at baseline were excluded from the analysis. PA related questions were analyzed using repeated measures ANOVA across the three times points (baseline vs. 6-month vs. 12-month follow-up) with weekly minutes of accelerometer MVPA as the dependent variable. Subsequent repeated measures mixed ANOVA was also conducted with meeting MVPA guidelines (meeting vs. not meeting) as the between-subjects variable, time as the within-subjects variable and weekly minutes of accelerometer MVPA as the dependent variable. Differences in MVPA change between groups were assessed by three one-way ANOVAs with group as the independent variable and MVPA change (baseline to 6-month change, 6-month to 12-month change, baseline to 12-month change) as the dependent variables and a Bonferroni correction applied to the three analysis to adjust for repeated analyses on the same data. For PA motivation and QoL questions repeated measures ANOVA were conducted with time as the within-subject variable. Repeated measures ANOVAs used polynomial contrasts to describe trends over time and one-way ANOVAs used post-hoc t-tests. All analyses assumptions were tested, and in instances of sphericity violations Greenhouse-Geisser or Huynh-Feldt corrections were used depending on epsilon values.

Given the overarching aim of Project MOVE was feasibility and the nature of this paper was to explore an estimate of effectiveness, a power calculation was not conducted, which is common practice amongst feasibility and pilot trails [37–39].

## Results

Table 1 presents the baseline characteristics for all Project MOVE participants. Self-report data were collected from 87 participants within 10 unique PA groups at baseline, 72 (82.7%) at 6-month follow-up and 60 (68.9%) at 12-month follow-up. The total sample at baseline (*n* = 87) included 71 BC survivors (82%), 3 other cancer survivors (4%), and 13 healthy support individuals (15%). At 6 months reasons for drop-out included: deterioration of health (*n* = 9), could not be reached (*n* = 2), not interested (*n* = 2), and death (n = 2). At 12 month follow-up, 12 participants did not attend data collection and did not provide a reason for this absence. Table 2 provides a brief description of the groups and PA activities undertaken by each group, inclusive of an approximate MET Range for each group based on the Compendium of Physical Activities [40]. At baseline, participants had a mean age of 58.8 ± 8.7 (mean ± SD) years and a mean BMI of 25.9 ± 4.8 kg/m^2^. The majority of participants were married or living with a life partner (*n* = 60, 69%) and had completed a college diploma or university degree (*n* = 56, 64.4%). Of the 71 BC survivors, 40 had been diagnosed with Stage 0 to Stage II breast cancer. One-way ANOVA showed those who had been diagnosed with BC did not significantly differ in their MVPA (M = 179.38 SD = 96.14) than those who had not been diagnosed with breast cancer at baseline (M = 189.13 SD = 96.14.

**Table 1:** Participant Demographics (*n* = 87)

Variable	Participant %, (n)
Age (years)^a^	
35–44	4.6 (4)
45–54	23 (20)
55–64	41.4 (36)
65–74	24.1 (21)
75–84	1.1 (1)
Ethnicity^b^	
White	94.3 (82)
Asian	3.4 (3)
Black	1.1 (1)
Education^c^	
High school or less	1.1 (1)
High school diploma	9.2 (8)
Some post-secondary without diploma or degree	19.5 (17)
College or technical diploma or certificate	39.1 (34)
University Degree	25.3 (22)
Other	4.6 (4)
Martial Status^d^	
Married or living with a life partner	69 (60)
Living alone	23 (20)
Widowed	6.9 (6)
Employment^e^	
Full time work	29.9 (26)
Part time work	14.9 (13)
Caring for family/managing household	4.6 (4)
Unemployed	2.3 (2)
Recovering from illness/disability	8 (7)
Retired	34.5 (30)
Other	4.6 (4)
BC staging	
Stage 0	6.9 (6)
Stage I	14.9 (13)
Stage II	24.1 (21)
Stage III	14.9 (13)
Stage IV	8 (7)
Unknown	12.6 (11)
BC treatment^f^	
Lymph or axillary node dissection	66.7 (58)
Radiotherapy	54 (47)
Chemotherapy	51.7 (45)
Lumpectomy	47.1 (41)
Reconstructive surgery	31 (27)
Hormonal Therapy	28.7 (25)
Single Mastectomy	28.8 (25)
Double Mastectomy	20.7 (18)
Other	4.6 (4)
Menopause status^g^	
Pre-menopausal	8 (7)
Going through menopause	10.3 (9)
Post-menopausal	65.5 (57)

Note. *BC* = Breast Cancer, a n = 5 participants did not report, b,c,d,e 1n = 1 participant did not report, f participants indicated 1 or more options, g 4n = 4 participants did not report

**Table 2:** Brief Description of Project MOVE Groups and Microgrant Activities

Groups	Microgrant Activities
1) Women on Weights (*n* = 5)	- Participants hired a certified exercise trainer to deliver weight training (e.g., free weights, resistance bands, weight machines) to participants. MET Range 3–6
2) Group Training (*n* = 10, 2 participants were healthy support individuals)	- Participants hired a certified exercise trainer to deliver group circuit training, which included strength training with equipment, body weight exercises, and cardio using treadmills, elliptical machines and stationary bikes. MET Range 3–8
3) Explore Movement (*n* = 8)	- Participants hired a certified exercise trainer to deliver a variety of activities including strength training, yoga, pilates, and low impact aerobics. MET Range 2–5
4) Move Anytime Anywhere (*n* = 12)	- Participants hired a certified exercise trainer to deliver a variety of activities including strength training with free weights and body weight and group walking/jogging. Met Range 3–8
5) Strive to Thrive (*n* = 12)	- Participants purchased Fitbits™ & free weights for each member of the group. Participants walked together and participated in low intensity weight training taught by a hired exercise trainer after each walk. MET Range 2–8
6) Spin Together (*n* = 8, 2 participants were healthy support individuals)	- Participants purchased and attended spin class together and participated in light yoga movements and stretching afterwards. MET Range 2–8
7) Fit Together (*n* = 12, 3 participants were healthy support individuals)	- Participants hired a certified exercise trainer to deliver a variety of activities including strength training with free weights and body weight. In addition they participated in group walking/jogging. MET Range 3–8
8) New Wave Warriors (*n* = 9, 4 participants were healthy support individuals)	- Participants hired a certified exercise trainer to deliver a variety of exercise options including “boot camp” style workouts, walking and hiking, and yoga. MET Range 3–9
9) iHealth (*n* = 6, 3 participants were other cancer survivors)	- Participants hired a certified exercise trainer to deliver a variety of exercise options including strength training with free weights, resistance bands, TRX, as well as group aerobic activities such as low impact aerobics. MET Range 3–6
10) Spin to Health (*n* = 5, 2 participants were healthy support individuals)	- Participants purchased and attended spin, barre, yoga and combination classes together. MET Range 2–8

Accelerometer data were collected from 88 participants at baseline, 69 (78.4%) at 6-month follow-up and 65 (73.8%) at 12-month follow-up.

### Physical activity

At baseline, one participant had insufficient accelerometer wear-time, resulting in valid data from 87 participants. At 6-month and 12-month follow-up, four participants had insufficient wear-time and had observations carried forward. At baseline 48 (54.5%) participants were meeting MVPA guidelines and 39 (44.3%) were not, at 6 months follow-up 56 (64.4%) were meeting MVPA guidelines and at 12 month follow-up 40 (46.0%) were meeting MVPA guidelines. Repeated measures ANOVA showed no significant differences between time points on weekly light activity (*F*(2,174) = 2.37, *p* = .096). Repeated measures ANOVA showed significant differences and a small to medium effect size between time points on weekly MPVA (*F*(2,170) = 3.62, *p* = .03, eta^2^ = .041). Polynomial contrasts showed a significant quadratic trend (*F*(1,85) = 6.32, *p* = .01). See Table 3 for means and standard deviations across time points.

**Table 3:** Weekly Minutes of PA, PA Motivation and QoL Means Scores Across Time Points

Variable	Baseline Mean (SD)	6-Month Mean (SD)	12-Month Mean (SD)	Effect Size Overall Interaction
Light Activity				
Overall	1802.48 (490.39)	1785.93 (540.12)	1700.43 (590.80)	0.006
Meeting MVPA	1904.89 (500.54)	1841.34 (553.34)	1735.45 (567.93)	
Not Meeting MVPA	1673.82 (451.21)	1716.31 (521.75)	1656.43 (623.01)	0.003
MVPA				
Overall	178.51 (115.85)	191.62 (112.92)	165.70 (115.05)	0.012
Meeting MVPA	264.46 (83.08)	252.73 (95.72)	226.90 (107.42)	
Not Meeting MVPA	74.92 (38.98)	117.97 (85.15)	91.92 (73.61)	0.02
BREQ				
Amotivation				
Overall	0.20 (0.51)	0.13 (0.38)	0.13 (0.37)	0.007
Meeting MVPA	0.05 (0.29)	0.01 (0.07)	0.02 (0.08)	
Not Meeting MVPA	0.39 (0.67)	0.29 (0.53)	0.28 (0.51)	0.002
External Regulation				
Overall	0.72 (0.87)	0.75 (0.88)	0.75 (0.91)	> 0.000
Meeting MVPA	0.58 (0.75)	0.52 (0.64)	0.56 (0.72)	
Not Meeting MVPA	0.89 (0.99)	1.04 (1.06)	0.99 (1.06)	0.002
Introjected Regulation				
Overall	2.12 (0.95)	1.98 (0.95)	1.89 (0.92)	0.008
Meeting MVPA	2.23 (0.97)	1.95 (0.96)	1.89 (0.98)	
Not Meeting MVPA	1.99 (0.87)	2.01 (0.96)	1.89 (0.85)	0.005
Identified Regulation				
Overall	3.18 (0.75)	3.25 (0.72)	3.27 (0.69)	0.004
Meeting MVPA	3.51 (0.52)	3.53 (0.53)	3.58 (0.51)	
Not Meeting MVPA	2.76 (0.77)	2.91 (0.80)	2.88 (0.70)	0.001
Integrated Regulation				
Overall	2.51 (1.12)	2.80 (1.03)	2.84 (1.09)	0.027
Meeting MVPA	3.09 (0.84)	3.14 (0.85)	3.36 (0.67)	
Not Meeting MVPA	1.82 (1.01)	2.39 (1.09)	2.23 (1.17)	0.014
Intrinsic Regulation				
Overall	2.91 (1.00)	3.03 (0.90)	3.05 (0.86)	0.006
Meeting MVPA	3.28 (0.78)	3.31 (0.69)	3.29 (0.72)	
Not Meeting MVPA	2.43 (1.07)	3.03 (0.9)	2.74 (0.95)	0.003
SF-36				
Physical Functioning				
Overall	78.66 (20.38)	86.65 (15.7)	85.02 (16.69)	0.028
Meeting MVPA	77.82 (22.41)	85.09 (18.86)	84.61 (19.23)	
Not Meeting MVPA	79.73 (17.71)	88.62 (10.31)	85.54 (13.00)	0.014
Role Functioning-Physical				
Overall	58.33 (44.30)	74.40 (40.22)	66.67 (42.75)	0.037
Meeting MVPA	59.04 (44.36)	64.36 (45.37)	62.23 (43.57)	
Not Meeting MVPA	57.43 (44.30)	87.16 (28.30)	72.30 (41.58)	0.001
Role Functioning-Emotional				
Overall	64.86 (41.65)	74.70 (38.46)	74.89 (40.67)	0.015
Meeting MVPA	61.23 (42.75)	63.77 (43.79)	63.04 (45.12)	
Not Meeting MVPA	69.37 (40.35)	88.29 (25.11)	87.39 (29.76)	0.009
General Health				
Overall	66.35 (20.37)	71.47 (18.65)	70.37 (19.58)	0.013
Meeting MVPA	65.32 (21.12)	69.25 (18.41)	68.22 (19.52)	
Not Meeting MVPA	67.66 (19.58)	74.28 (18.16)	73.11 (19.59)	0.001
Social Functioning				
Overall	73.96 (26.83)	83.18 (23.08)	80.36 (24.26)	0.027
Meeting MVPA	79.52 (26.25)	85.37 (22.47)	84.31 (23.38)	
Not Meeting MVPA	66.89 (26.22)	80.40 (23.85)	75.33 (24.73)	0.004

Mixed ANOVA further indicated a significant main effect and small effect size between time points on weekly MVPA (*F*(2,168) = 3.84, *p* = .02, eta^2^ = .012), but also showed a significant interaction and small effect size between those meeting MVPA guidelines at baseline and those not on weekly MVPA between time points (*F*(2,168) = 5.61, *p* = .004, eta^2^ = .02). Polynomial contrasts showed a significant increasing linear trend (*F*(1,84) = 7.55, *p* = .007). Descriptive statistics can be seen in Table 3.

### Change in MVPA by group

**Fig. 1:**
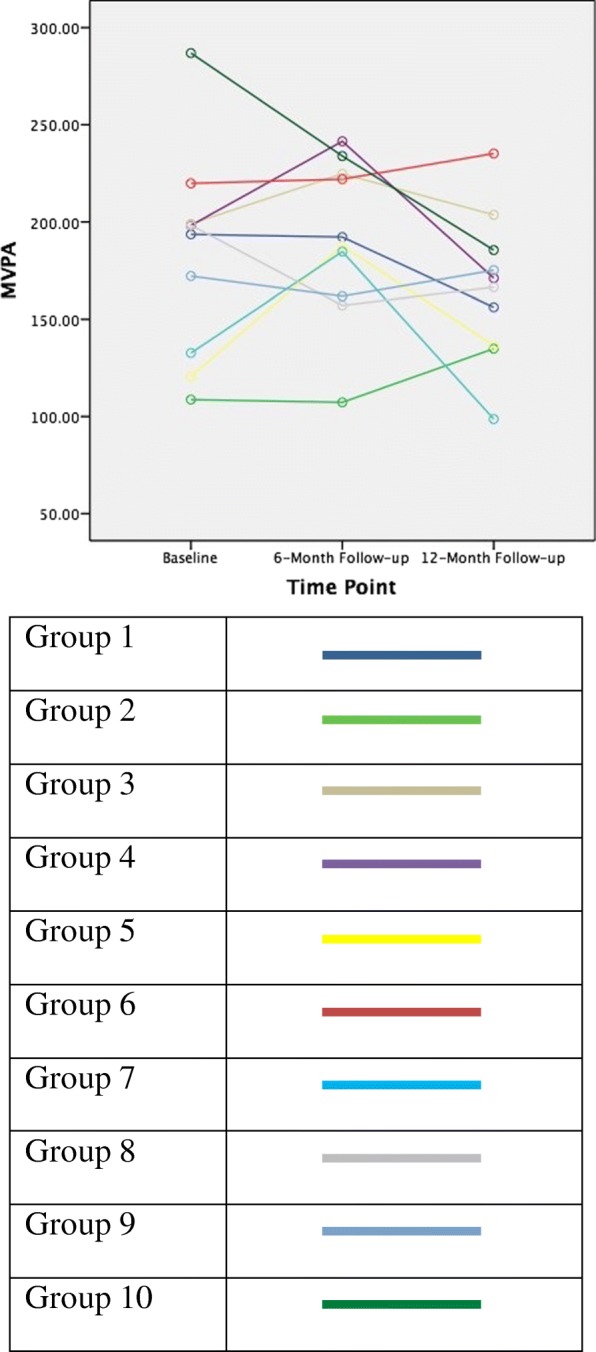
MVPA Means by Time Point by Group

One-way ANOVA with group as the independent variable and MVPA change between baseline and 6-month follow up showed no significant group differences (*F*(9,85) = 1.89, *p* = .066, eta^2^ = .183). One-way ANOVA with group as the independent variable and MVPA change between 6-month and 12-month follow up showed no significant group differences (*F*(9,85) = 1.86, *p* = .07, eta^2^ = .177). One-way ANOVA with group as the independent variable and MVPA change between baseline and 12-month follow up showed no significant group differences (*F*(9,85) = 1.09, *p* = .382, eta^2^ = .114). Descriptive statistics by group can be seen in Table 4 and MVPA means by time point by group can be seen in Fig. 1.

**Table 4:** Mean Change in MVPA Between Time Points by Group

Group	N	Mean Change in MVPA (SD); 95% Confidence Interval		
		Baseline vs 6 – Month	6-Month vs 12-Month	Baseline vs. 12 - Month
1	5	−1.4 (36.6); −46.9 – 44.1	−36 (39.7); −85.3 – 13.3	−37.4 (70.1); −124.5 – 49.7
2	10	−1.4 (72.9); −53.6 – 50.8	27.6 (55.3); − 12.0 – 67.2	26.2 (113.9); −55.3 – 107.7
3	8	25.7 (71.2); −33.8 – 85.3	−21.1 (45.2); −58.9 – 16.7	4.6 (91.8); −72.2 – 81.4
4	12	43.3 (59.2); 5.6–81.0	−70.4 (119.5); − 146.4 – 5.54	−27.1 (84.2); −80.6 – 26.4
5	12	67.1 (67.0); 5.1–129.1	−3.7 (164.3); − 141.1 – 133.6	15.8 (74.4); − 53.0 – 84.7
6	8	2.2 (112.5); −69.3 – 73.7	13.1 (59.1); −24.4 – 50.8	15.3 (116.5); − 58.7 – 89.3
7	12	52.0 (80.7); 0.7–103.4	−86.1 (86.2); − 140.9 - -31.3	− 34.0 (43.0); −61.4 - -6.6
8	9	−41.2 (73.9); −98.1 – 15.6	9.33 (95.5); − 64.1 – 82.8	−31.9 (109.2); − 115.8 – 52.1
9	6	− 10.3 (37.6); − 49.8 – 29.2	16.0 (73.5); −52.0 – 84.0	3.0 (94.2); −95.9 – 101.9
10	5	−53.0 (125.4); − 208.7 – 102.7	−48.4 (104.8); − 178.5 – 81.7	−101.4 (127.8); − 260.1 – 57.3
Total	87	13.1 (83.0); − 4.6 – 30.9	− 21.2 (96.3); − 41.6 - -0.8	−12.8 (95.1); − 33.2 – 7.6

### Physical activity motivation

Repeated measures mixed ANOVAs, with Huynh-Feldt corrections when applicable, were conducted to assess changes in motivation regulations across time points. There was a significant change and very small effect size for intrinsic regulation (F(2, 168) = 3.95, *p* = .02, eta^2^ = .0006) and a significant interaction between those meeting and those not meeting MVPA guidelines at baseline (F(2,168) = 3.41, *p* = .035, eta^2^ = .003). Polynomial contrasts showed a significant linear trend (F(1,84) = .98, p = .02). Descriptive statistics can be seen in Table 3.

### Quality of life

Repeated measure mixed ANOVA shows significant differences and small effect size across time points on physical functioning (*F*(1.32,110.36) = 17.83, *p* < .001, eta^2^ = .028), with no significant interaction, polynomial contrasts show significant linear (*F*(1,82) = 14.15, *p* < .001) and quadratic trends (*F*(1,82) = 26.16, *p* < .001). Role functioning-physical shows significant differences and small effect size across time points on role functioning-physical (*F*(2,164) = 7.82, *p* = .001, eta^2^ = .037), mediated by a significant interaction between those meeting and those not meeting MVPA guidelines at baseline (*F*(2,164) = 3.79, *p* = .024, *p* = .024, eta^2^ = .014), a significant quadratic trend was found (*F*(1,82) = 6.78, *p* = .01). Role-functioning emotional shows significant differences and small effect size across time points (*F*(2,162) = 3.86, *p* = .023, eta^2^ = .015) with no significant interaction, and a significant liner trend (*F*(1,81) = 4.8, *p* = .03). Social functioning shows significant differences and small effect size across time points (*F*(1.71, 140.54) = 7.80, *p* = .001, eta^2^ = .027) with no significant interaction and significant linear (*F*(1,82) = 5.87, *p* = .019,) and quadratic trends (*F*(1,82) = 10.59, *p* = .002). General health shows significant differences and small effect size across time points (*F*(1.68, 137.90) = 6.27, *p* = .004, eta^2^ = .013) with no significant interaction, and significant linear (*F*(1,82) = 5.42, *p* = .02) and quadratic trends (*F*(1,82) =7.84, *p* = .006). Descriptive statistics are presented in Table 3.

## Discussion

Project MOVE was designed as an innovative strategy that encouraged groups of female BC survivors to come together and tailor a PA program to their specific needs and interests. Results showed that mean PA significantly increased at 6-month follow-up. However, the greater PA levels were not maintained at 12 months. Moreover, a statistically significant linear trend was observed for those not meeting guidelines at baseline, in that they continued to increase their PA at 6 months and 12 months, compared to baseline. Those meeting guidelines at baseline did not significantly improve PA levels but did maintain PA above the recommend guidelines (≥150 min per week) at 6 and 12 month follow-ups. Intrinsic motivation increased over time, and participants also reported statistically significant improvements in physical functioning, physical health limitations, emotional health limitations, and general health at 6-month follow-up. At the 12-month follow-up, statistically significant changes in physical functioning and general health were noted. In combination, these findings provide promising evidence for Project MOVE as an effective strategy to increase PA and improve PA motivations and QoL among breast cancer survivors.

An important finding of the current study is that among participants not meeting guidelines at baseline, their PA significantly increased on average by 43 min per week of MVPA at the 6-month follow-up and 20 min per week at the 12 month follow-up. Given that more than 70% of survivors in the literature are reported to be inactive post-treatment [12–14], these findings suggest that Project MOVE may be an effective strategy for initiating PA engagement among a substantial, at-risk subset of BC survivors. The approach in which participants were provided with an opportunity to develop and implement their own PA initiatives may have contributed to this increase. Previous reports have indicated that low PA engagement amongst BC survivors is often due to a lack of confidence and uncertainty with how to perform certain exercises, and fear of injury due to the physical limitations common to BC survivors (e.g., lymphedema, fatigue) [41–43]. The sense of autonomy associated with the Project MOVE model provided these women with an opportunity to optimize their own strengths in developing an initiative that addressed specific BC related limitations and met their own needs and interests, thus aiding with issues surrounding PA confidence and efficacy.

PA behaviors adopted by participants while taking part in Project MOVE did not change over time. In the context of Project MOVE, some of the PA initiatives developed may be more sustainable than others. For example, in situations where the microgrant money was used to take part in paid fitness classes, participation may decline once funding runs out, whereas groups that purchased activity trackers (e.g., FitBits™) may have greater potential for sustainability. One such strategy that may assist with the increase and maintenance of PA post-intervention is the use of a tapered intervention design, in which participants are transitioned to other community based programs and/or home-based exercise. For instance, group leaders could provide participants with information on free opportunities to be active in the community and additional resources encouraging participants to continue to be active together (i.e., “take home” information packages with examples and instructions on how to be active at home and with friends). Future deliveries of Project MOVE may also wish to consider the potential for PA maintenance as a criterion during the grant application and review process.

Among those meeting PA guidelines at baseline (average of 264 mins of MVPA per week), there were no statistically significant changes in weekly minutes of PA. Mean minutes of MVPA in this group were already well above those required for health benefits and Project MOVE may only contributed to the maintaining of their weekly PA levels. Results support Project MOVE, and one could speculate that the autonomy for the women to design and collectively tailor PA programs and initiatives to meet their needs and preferences (as provided by the Project MOVE Model) allows greater opportunity for program satisfaction and enjoyment, which has been previously known to influence PA participation and maintenance [44–46]. However, it is important to note that a further increase in PA, of any magnitude (i.e., intensity, frequency, duration), could provide even greater health benefit and thus it may be beneficial to provide some assistance around program design for these particular individuals. Although the autonomy to design their own program or initiative proved successful, it may be that these individuals require additional guidance and advice to ‘take it to the next level’ to elicit greater improvements in PA behavior and overall health.

In addition to the health benefits associated with the maintenance of PA above and beyond the recommended guidelines, it is important to note these findings make a valuable contribution to the limited empirical evidence concerning the long-term (at least 3 months post-intervention follow-up) maintenance of PA behavior change [47–49] and the utilization of objective measures to assess long-term PA outcomes [50, 51]. A recent review on the maintenance of PA behavior change interventions of cancer patients reported that only 10 out of 63 trials assessed post-intervention maintenance, and of these 10 only 4 achieved maintenance success, defined as at least 3 months post-intervention follow-up [47]. Furthermore, there is a documented need for greater use of objective measures of PA (i.e., accelerometry) over and above self-report measures, in order to provide more detailed and accurate information about PA behaviors within this sub-population [47, 50–52].

Given that motivation is a key predictor of PA [53, 54], different motivation regulations were assessed for changes during Project MOVE. Overall, intrinsic motivation significantly increased over time. There were also greater increases in intrinsic motivation for women who were not meeting PA guidelines at baseline. This finding is particularly important since intrinsic motivation is critical to PA adherence, effort, and participation among cancer survivors [55, 56]. Based on Self-Determination Theory [57], it may be that Project MOVE fostered the basic psychological needs of autonomy and relatedness given the grassroots, group-based, program. Additionally, perceptions of competence may have been fostered with feedback provided among group members and role modeling that was conducive the program. While speculative, future research initiatives should be focused on the theoretical mechanisms that explain the increases in intrinsic motivation over time, and a longer term follow-up would provide foundational evidence on how intrinsic motivation is associated with physical activity behavior given that intrinsic motivation may be the most important motivation regulation for exercise adherence [53].

Findings from the current study align with many previous BC studies that have also indicated improvements to the QoL subscales (physical functioning, role function-physical, role functioning-emotional, and general health) post PA intervention [10, 58–61]. Improvements in QoL are important psychosocial adjustments following a PA intervention. The current study was focused on exploring changes in QoL, yet future research directions are needed to identify and explore mechanisms that help to explain these improvements in physical functioning and health. While not tested in the current study, these improvements may be explained by increases in PA, and social support from hired trainers and other women in the Project MOVE groups [62–64]. Furthermore, women may have developed self-confidence and self-efficacy with performing PA [65–67] that helped foster activity and improvements in QoL. Although these findings indicate statistically significant improvements, these differences were small and likely not clinically meaningful, warranting further investigation concerning these mechanisms.

### Strengths and limitations

Unique to Project MOVE was the use of the microgrants and a financial incentive. The program was designed to encourage BC survivors to work together to create a strategy that met their specific needs and interests. Contrary to contemporary PA interventions that provide a more structured and pre-determined design, the Project MOVE model represents a highly flexible and adaptable community-based model that requires relatively low follow-up. Additionally, the use of accelerometers as an objective measure of PA as well as a long follow-up period is also a strength of this study. Previous research with this population has often utilized self-report measures of PA and shorter follow-up periods (< 6 months). Moreover, this is the first study to evaluate PA behavior change within in a microgrant model. Previous research has been limited to descriptive research and process evaluations concerning the uptake and implementation of such models [15–18].

Eligibility was modified to include additional women who wished to participate but were not BC survivors. Although this limits the generalizability of these findings, the inclusion of non-BC female support (i.e., friends and/or relatives) is an interesting strategy to increase participation among BC survivors. As many women may be hesitant to participate alone, allowing friends and/or relatives to join may help to engage more survivors as well as provide an opportunity to strengthen existing support structures. Furthermore, this may also act as a prevention strategy for those who have never been diagnosed with cancer as these friends and/relatives would be exposed to the information and knowledge presented during each session concerning BC and physical and psychological benefits of PA. This study was also limited in terms of diversity, thus further affecting generalizability. The lack in gender, ethnic and social diversity of participants limits representation of the entire BC survivor population. However, it should be noted that the organic nature of the microgrant model (i.e., being able to develop your own intervention) does provide an opportunity for transferability to other cancer, disease, and general populations.

Further, there are also a number of study design and methodological limitations. First, given the exploratory nature of the study and aim of this specific paper (i.e., estimate of effect) a power calculation was not conducted. Second, issues surrounding the of use of accelerometers as a measure of PA are also identified, specifically concerning specificity and sensitivity of accelerometers in differentiating between different modes of physical activity (e.g., habitual activity vs. planned intervention activity) and identifying activity that is undetectable by accelerometers (e.g., flexibility training or some strength training). Third, the use of “last observation carried forward” has limitations [68], however the majority of participants with missing data had only one time point of data collected, making growth curve analysis inappropriate. Fourth, multilevel modeling was not used to analyze the data because we were interested in broad group-level trends rather than how individuals responded to the specific interventions. As such, the clustering of women within groups and over time was not accounted for statistically. This being said, future research could focus on the specific within- and between-person effects of microgrant frameworks on PA, motivation, and QoL. Lastly, we did not collect information concerning physical activity dose, adherence, or intervention variations specific to each group, which would be beneficial with understanding the meaningfulness (interpreted in the context of design, power, and analytical framework) of each separate group intervention.

## Conclusion

The findings from this exploratory research support the use of a microgrants program for increasing PA and QoL, with particular benefits for breast cancer survivors who are not active. However, a number of limitations have been identified and thus further pilot work is needed to address some of these limitations, followed by a fully powered RCT to test intervention effectiveness as well as identify strategies to promote PA maintenance over time.

## Abbreviations

BC: Breast cancer
MVPA: Moderate vigorous physical activity
PA: Physical activity
QoL: Quality of life
RCT: Randomized control trial
SD: Standard deviations

## References

[1] Ferlay J, Soerjomataram I, Evrik M, Dikshit R, Eser S, Mathers C, Rebelo M, Parkin D, Forman D, Bray F: Cancer incidence and mortality worldwide. In: *IARC CancerBase No11.* Lyon, FR: International Agency for Research on Cancer; 2014.

[2] Canadian Cancer Society’s Advisory Committee on Cancer Statistics: Canadian Cancer Statistics 2017. In*.* Toronto, ON: Canadian Cancer Society 2017.

[3] Cleeland CS, Zhao F, Chang VT, Sloan JA, O'Mara AM, Gilman PB, Weiss M, Mendoza TR, Lee JW, Fisch MJ (2013). The symptom burden of cancer: evidence for a core set of cancer-related and treatment-related symptoms from the eastern cooperative oncology group symptom outcomes and practice patterns study. Cancer.

[4] Demark-Wahnefried W, Aziz NM, Rowland JH, Pinto BM (2005). Riding the crest of the teachable moment: promoting long-term health after the diagnosis of cancer. J Clin Oncol.

[5] Ibrahim EM, Al-Homaidh A (2011). Physical activity and survival after breast cancer diagnosis: meta-analysis of published studies. Med Oncol.

[6] Schmitz KH, Courneya KS, Matthews C, Demark-Wahnefried W, Galvao DA, Pinto BM, Irwin ML, Wolin KY, Segal RJ, Lucia A (2010). American College of Sports Medicine roundtable on exercise guidelines for cancer survivors. Med Sci Sports Exerc.

[7] Kim J, Choi WJ, Jeong SH (2013). The effects of physical activity on breast cancer survivors after diagnosis. J Cancer Prev.

[8] Demark-Wahnefried W, Rogers LQ, Alfano CM, Thomson CA, Courneya KS, Meyerhardt JA, Stout NL, Kvale E, Ganzer H, Ligibel JA (2015). Practical clinical interventions for diet, physical activity, and weight control in cancer survivors. CA Cancer J Clin.

[9] Sabiston CM, Brunet J, Burke S (2012). Pain, movement, and mind: does physical activity mediate the relationship between pain and mental health among survivors of breast cancer. Clin J Pain.

[10] Shin WK, Song S, Jung SY, Lee E, Kim Z, Moon HG, Noh DY, Lee JE (2017). The association between physical activity and health-related quality of life among breast cancer survivors. Health Qual Life Outcomes.

[11] Lahart IM, Metsios GS, Nevill AM, Carmichael AR (2015). Physical activity, risk of death and recurrence in breast cancer survivors: a systematic review and meta-analysis of epidemiological studies. Acta Oncol.

[12] Lynch BM, Dunstan DW, Healy GN, Winkler E, Eakin E, Owen N: Objectively measured physical activity and sedentary time of breast cancer survivors, and associations with adiposity: findings from NHANES(2003-2006). Cancer Causes Control 2010, 21(2):283–288. 10.1007/s10552-009-9460-6 19882359

[13] Blanchard CM, Courneya KS, Stein K: Cancer survivors' adherence to lifestyle behavior recommendations and associations with health-related quality of life: results from the American Cancer Society's SCS-II. J Clin Oncol 2008, 26(13):2198–2204. 10.1200/JCO.2007.14.6217 18445845

[14] Mason C, Alfano CM, Smith AW, Wang CY, Neuhouser ML, Duggan C, Bernstein L, Baumgartner KB, Baumgartner RN, Ballard-Barbash R (2013). Long-term physical activity trends in breast cancer survivors. Cancer Epidemiol Biomark Prev.

[15] Bobbitt-Cooke M: Energizing community health improvement: the promise of microgrants. Prev Chronic Dis 2005, 2Spec.no:A16. PMC1459464 16263049

[16] Caperchione C, Mummery WK, Joyner K (2010). WALK community Grants scheme: lessons learned in developing and administering a health promotion microgrants program. Health Promot Pract.

[17] Collie-Akers V, Schultz JA, Carson V, Fawcett SB, Ronan M (2009). Evaluating mobilization strategies with neighborhood and faith organizations to reduce risk for health disparities. Health Promot Pract.

[18] Schmidt M, Plochg T, Harting J, Klazinga NS, Stronks K (2009). Micro grants as a stimulus for community action in residential health programmes: a case study. Health Promot Int.

[19] Pullen T, Bottorff JL, Sabiston CM, Campbell KL, Eves ND, Ellard SL, Gotay C, Fitzpatrick K, Sharp P, Caperchione CM. Utilizing RE-AIM to examine the translational potential of project MOVE, a novel intervention for increasing physical activity levels in breast cancer survivors. Transl Behav Med. 2018. 10.1093/tbm/iby081 30060250

[20] Pullen T, Sharp P, Bottorff JL, Sabiston CM, Campbell KL, Ellard SL, Gotay C, Fitzpatrick K, Caperchione CM (2018). Acceptability and satisfaction of project MOVE: a pragmatic feasibility trial aimed at increasing physical activity in female breast cancer survivors. Psychooncology.

[21] Caperchione CM, Sabiston CM, Clark MI, Bottorff JL, Toxopeus R, Campbell KL, Eves ND, Ellard SL, Gotay C (2016). Innovative approach for increasing physical activity among breast cancer survivors: protocol for project MOVE, a quasi-experimental study. BMJ Open.

[22] Defining Cancer Survivorship [http://www.canceradvocacy.org/news/defining-cancer-survivorship/].

[23] Troiano RP, Berrigan D, Dodd KW, Masse LC, Tilert T, McDowell M (2008). Physical activity in the United States measured by accelerometer. Med Sci Sports Exerc.

[24] Freedson PS, Melanson E, Sirard J (1998). Calibration of the computer science and applications, Inc. accelerometer. Med Sci Sports Exerc.

[25] Shiroma EJ, Cook NR, Manson JE, Buring JE, Rimm EB, Lee IM (2015). Comparison of self-reported and accelerometer-assessed physical activity in older women. PLoS One.

[26] Livingston PM, Craike MJ, Salmon J, Courneya KS, Gaskin CJ, Fraser SF, Mohebbi M, Broadbent S, Botti M, Kent B et al: Effects of a clinician referral and exercise program for men who have completed active treatment for prostate cancer: a multicenter cluster randomized controlled trial (ENGAGE). Cancer 2015, 121(15):2646–2654. 10.1002/cncr.29385 PMC4654333 25877784

[27] Fukuoka Y, Haskell W, Vittinghoff E: New insights into discrepancies between self-reported and accelerometer-measured moderate to vigorous physical activity among women - the mPED trial. BMC Public Health 2016, 16(1):761. 10.1186/s12889-016-3348-7 PMC4982411 27514368

[28] Tucker JM, Welk GJ, Beyler NK, Kim Y (2016). Associations between physical activity and metabolic syndrome: comparison between self-report and Accelerometry. Am J Health Promot.

[29] Duncan LR, Hall CR, Wilson PM, Jenny O (2010). Exercise motivation: a cross-sectional analysis examining its relationships with frequency, intensity, and duration of exercise. Int J Behav Nutr Phys Act.

[30] Sicilia A, Saenz-Alvarez P, Gonzales-Cutre D, Ferris R. Exercise motivation and social physique anxiety in adolescents. Psychologica Belgica. 2014:54.

[31] Deci EL, Ryan RM (1985). Intrinsic motivation and self-determination in human behavior.

[32] Ryan RM, Deci EL: The "what" and "why" of goal pursuits: human needs and the self-determination of behavior. Psychol Inq 2001, 11:227–268.

[33] Wilson PM, Sabiston CM, Mack DM, Blanchard CM (2013). On the nature and function of scoring protocols used in exercise motivation research: an empirical study of the behavioral regulation in exercise questionnaire. Psych Sport Exer.

[34] Hays RD, Morales LS: The RAND-36 measure of health-related quality of life. Ann Med 2001, 33(5):350–357. 10.3109/07853890109002089 11491194

[35] Ware JE, Jr., Sherbourne CD: The MOS 36-item short-form health survey (SF-36). I. Conceptual framework and item selection. Med Care 1992, 30(6):473–483. 1593914

[36] Hays RD, Sherbourne CD, Mazel RM (1993). The RAND 36-item health survey 1.0. Health Econ.

[37] Billingham SA, Whitehead AL, Julious SA (2013). An audit of sample sizes for pilot and feasibility trials being undertaken in the United Kingdom registered in the United Kingdom clinical research network database. BMC Med Res Methodol.

[38] Arain M, Campbell MJ, Cooper CL, Lancaster GA: What is a pilot or feasibility study? A review of current practice and editorial policy. BMC Med Res Methodol 2010, 10:67. 10.1186/1471-2288-10-67 PMC2912920 20637084

[39] Bell ML, Whitehead AL, Julious SA (2018). Guidance for using pilot studies to inform the design of intervention trials with continuous outcomes. Clin Epidemiol.

[40] Ainsworth BE, Haskell WL, Herrmann SD, Meckes N, Bassett DR, Tudor-Locke C, Greer JL, Vezina J, Whitt-Glover MC, Leon AS (2011). 2011 compendium of physical activities: a second update of codes and MET values. Med Sci Sports Exerc.

[41] Brunet J, Taran S, Burke S, Sabiston CM (2013). A qualitative exploration of barriers and motivators to physical activity participation in women treated for breast cancer. Disabil Rehabil.

[42] Rogers LQ, Courneya KS, Verhulst S, Markwell SJ, McAuley E (2008). Factors associated with exercise counseling and program preferences among breast cancer survivors. J Phys Act Health.

[43] McAuley E, Blissmer B (2000). Self-efficacy determinants and consequences of physical activity. Exerc Sport Sci Rev.

[44] Hagberg LA, Lindahl B, Nyberg L, Hellenius ML (2009). Importance of enjoyment when promoting physical exercise. Scand J Med Sci Sports.

[45] Dunton GF, Vaughan E (2008). Anticipated affective consequences of physical activity adoption and maintenance. Health Psychol.

[46] Mullen SP, Olson EA, Phillips SM, Szabo AN, Wojcicki TR, Mailey EL, Gothe NP, Fanning JT, Kramer AF, McAuley E (2011). Measuring enjoyment of physical activity in older adults: invariance of the physical activity enjoyment scale (paces) across groups and time. Int J Behav Nutr Phys Act.

[47] Spark LC, Reeves MM, Fjeldsoe BS, Eakin EG (2013). Physical activity and/or dietary interventions in breast cancer survivors: a systematic review of the maintenance of outcomes. J Cancer Surviv.

[48] Short CE, James EL, Stacey F, Plotnikoff RC (2013). A qualitative synthesis of trials promoting physical activity behaviour change among post-treatment breast cancer survivors. J Cancer Surviv.

[49] Demark-Wahnefried W, Morey MC, Sloane R, Snyder DC, Miller PE, Hartman TJ, Cohen HJ (2012). Reach out to enhance wellness home-based diet-exercise intervention promotes reproducible and sustainable long-term improvements in health behaviors, body weight, and physical functioning in older, overweight/obese cancer survivors. J Clin Oncol.

[50] Ritvo P, Obadia M, Santa Mina D, Alibhai S, Sabiston C, Oh P, Campbell K, McCready D, Auger L, Jones JM (2017). Smartphone-enabled health coaching intervention (iMOVE) to promote long-term maintenance of physical activity in breast Cancer survivors: protocol for a feasibility pilot randomized controlled trial. JMIR Res Protoc.

[51] Moore SC, Lee IM, Weiderpass E, Campbell PT, Sampson JN, Kitahara CM, Keadle SK, Arem H, Berrington de Gonzalez A, Hartge P et al: Association of Leisure-Time Physical Activity with Risk of 26 types of Cancer in 1.44 million adults. JAMA Intern Med 2016, 176(6):816–825. 10.1001/jamainternmed.2016.1548 PMC5812009 27183032

[52] Schrack J, Gresham G, Wanigatunga AA (2018). Understanding physical activity in Cancer patients and survivors: new methodology, new challenges, and new Opportunitites. Cold Spring Harb Mol Case Stud.

[53] Teixeira PJ, Carraca EV, Markland D, Silva MN, Ryan RM (2012). Exercise, physical activity, and self-determination theory: a systematic review. Int J Behav Nutr Phys Act.

[54] Ryan RM, Williams GC, Patrick H, Deci EL (2009). Self-determination theory and physical activity: the dynamics of motivation in development and wellness. Hell J Psychol.

[55] Milne HM, Wallman KE, Guilfoyle A, Gordon S, Corneya KS (2008). Self-determination theory and physical activity among breast cancer survivors. J Sport Exerc Psychol.

[56] Wilson PM, Blanchard CM, Nehl E, Baker F (2006). Predicting physical activity and outcome expectations in cancer survivors: an application of self-determination theory. Psychooncology.

[57] Deci EL, Ryan RM (2002). Handbook of Slef-Determination Research.

[58] McNeely ML, Campbell KL, Rowe BH, Klassen TP, Mackey JR, Courneya KS (2006). Effects of exercise on breast cancer patients and survivors: a systematic review and meta-analysis. Cmaj.

[59] Fong DY, Ho JW, Hui BP, Lee AM, Macfarlane DJ, Leung SS, Cerin E, Chan WY, Leung IP, Lam SH (2012). Physical activity for cancer survivors: meta-analysis of randomised controlled trials. BMJ.

[60] Kendall AR, Mahue-Giangreco M, Carpenter CL, Ganz PA, Bernstein L (2005). Influence of exercise activity on quality of life in long-term breast cancer survivors. Qual Life Res.

[61] Harder H, Parlour L, Jenkins V (2012). Randomised controlled trials of yoga interventions for women with breast cancer: a systematic literature review. Support Care Cancer.

[62] Barber FD (2012). Social support and physical activity engagement by cancer survivors. Clin J Oncol Nurs.

[63] Ungar N, Wiskemann J, Weibmann M, Knoll A, Steindorf K, Sieverding M: Social support and social control in the context of cancer patients' exercise: a pilot study. Health Pyschol Open 2016:1–11. 10.1177/2055102916680991 PMC5546267 28815053

[64] Rogers LQ, Markwell S, Hopkins-Price P, Vicari S, Courneya KS, Hoelzer K, Verhulst S (2011). Reduced barriers mediated physical activity maintenance among breast cancer survivors. J Sport Exerc Psychol.

[65] McAuley E, Konopack JF, Morris KS, Motl RW, Hu L, Doerksen SE, Rosengren K: Physical activity and functional limitations in older women: influence of self-efficacy. J Gerontol B Psychol Sci Soc Sci 2006, 61(5):P270–P277. 10.1093/geronb/61.5.p270 16960230

[66] Lapier TK, Cleary K, Kidd J (2009). Exercise self-efficacy habitual physical activity, and fear of falling in patients with coronary heart disease. Cardiopulm Phys Ther J.

[67] Phillips SM, McAuley E (2014). Physical activity and quality of life in breast cancer survivors: the role of self-efficacy and health status. Psychooncology.

[68] Streiner D, Geddes J (2001). Intention to treat analysis in clinical trials when there are missing data. Evid Based Ment Health.

